# A Higher Frequency of CD4^+^CXCR5^+^ T Follicular Helper Cells in Adult Patients with Minimal Change Disease

**DOI:** 10.1155/2014/836157

**Published:** 2014-08-27

**Authors:** Nan Zhang, Pingwei Zhao, Amrita Shrestha, Li Zhang, Zhihui Qu, Mingyuan Liu, Songling Zhang, Yanfang Jiang

**Affiliations:** ^1^Key Laboratory of Zoonosis Research, Ministry of Education, The First Hospital of Jilin University, Changchun 130021, China; ^2^Department of Pediatrics, First Affiliated Hospital of Jiamusi University, Jiamusi 154002, China; ^3^Jiangsu Co-Innovation Center for Prevention and Control of Important Animal Infectious Diseases and Zoonoses, Yangzhou 225009, China

## Abstract

*Background*. T follicular helper (TFH) cells are involved in the humoral immune responses. This study is aimed at examining the frequencies of different subsets of CD4^+^CXCR5^+^ TFH cells in adult patients with minimal change disease (MCD). *Methods*. A total of 27 patients and 14 healthy controls (HC) were characterized for the levels of sera cytokines, inducible T-cell costimulator (ICOS), and programmed death 1 (PD-1) of positive TFH cells by flow cytometry. The level of sera IL-21 was examined; 24 h urinary protein and eGFR were calculated. The potential correlation between the frequency of different subsets of TFH cells and the values of clinical measures in MCD patients were analyzed. *Results*. The frequency of circulating CD4^+^CXCR5^+^, CD4^+^CXCR5^+^ICOS^+^, and CD4^+^CXCR5^+^PD-1^+^ TFH cells and the levels of sera IL-17A, IFN-*γ*, IL-2, IL-10, IL-4, and IL-21 were significantly higher in MCD patients (*P* < 0.05) than that in the HC group. Furthermore, the percentages of circulating CD4^+^CXCR5^+^ TFH cells were negatively correlated with the values of eGFR (*r* = −0.4849, *P* < 0.05) and the percentages of CD4^+^CXCR5^+^PD-1^+^ TFH cells were correlated positively with the levels of serum IL-21 (*r* = 0.6137, *P* < 0.05) and 24 h urinary protein (*r* = 0.1410, *P* < 0.05) in those patients. Also, the percentages of CD4^+^CXCR5^+^ICOS^+^ TFH cells were correlated positively with the levels of serum IL-21 (*r* = 0.6201, *P* < 0.05) and 24 h urinary protein (*r* = 0.7519, *P* < 0.05). Following standard therapies, the percentages of circulating CD4^+^CXCR5^+^, CD4^+^CXCR5^+^PD-1^+^, and CD4^+^CXCR5^+^ICOS^+^ TFH cells and the levels of serum IL-21 were significantly reduced, but the levels of serum IL-4 and IL-10 were increased (*P* < 0.05). *Conclusions*. A higher frequency of CD4^+^CXCR5^+^ TFH cells that existed in adult patients with MCD could be new target for intervention of MCD.

## 1. Introduction 

Minimal change disease (MCD) is the major cause of the nephritic syndrome (NS) in children [1, 2], accounting for 70 to 90% in children (below 10 years old) whereas in adults MCD is found in 10 to 15% of cases with primary NS [3]. The glomeration appears normal on kidney biopsy under light microscopy. This kind of kidney disease can self-subside and is sensitive to hormone drugs with high cure rate of almost 90%. But adult MCD relapses are frequent and may range from 62.3 to 73.1% [4].

The underlying cause for MCD is unclear, but it is often preceded by an infection or an allergic reaction [3]. In 1974, Shalhoub [5] first proposed that MCD is associated with T-cell dysfunction and speculated that T cell produces one or more permeability factors. In recent years, more researches have been devoted to such questions. However, it is still unclear how different types of CD4^+^ helper T cells regulate the pathogenesis of MCD.

Studies have revealed that patients with MCD have a predominant ongoing T-helper cell type 2 (Th2) immune response [6, 7]. CXCR5^+^CD4^+^ follicular helper T (TFH) cells may be crucial for regulating B-cell activation and germinal center formation, enhancing humoral responses. The most accurate definition of TFH cells relates to their function that migrates to follicles and interacts with B cells to support B-cell differentiation. However, it is difficult to isolate cells from anatomically discrete regions of lymphoid tissues. For this reason, TFH cells are more commonly defined on their surface phenotype [8]. However, there are clear differences between CD4^+^CXCR5^+^ T cells in the blood and those in the tonsils. For instance, CD4^+^CXCR5^+^ T cells in the blood do not express BCL-6 and their expression of ICOS and PD-1 is substantially lower than that of TFH cells [9, 10]. Human circulating CD4^+^CXCR5^+^ T cells have some features of TFH cells. And some researchers think it might be memory TFH cells. So we usually use blood CXCR5^+^CD4^+^ T cells to study TFH cells [11]. These molecules promote TFH development and T- and B-cell interaction [12]. PD-1 is a member of CD28/CTLA-4 coreceptor family that delivers inhibitory signals to T cells, ICOS is the other member of the same coreceptor family that provides costimulation of T cells [13, 14], and IL-21 is critical for the formation of germinal centers and the development of TFH cells [15, 16]. Increased numbers of circulating TFH cells and aberrant activation of TFH cells have been associated with the development of autoimmune diseases [12]. However, how TFH cells are associated with different stages of differentiated B cells in the pathogenesis of MCD and whether TFH cells participate in the pathogenesis of MCD is not fully understood. Currently, patients with MCD are usually treated with hormone drugs and immunosuppressant. These patients are also treated with antihypertensive drugs, such as angiotensin-converting enzyme (ACE) inhibitors and angiotensin II receptor antagonists, and thrombolytic drugs, such as aspirin or dipyridamole. However, it is unclear how these therapeutic strategies affect different subsets of TFH cells and immune measures in patients with MCD.

In our study, we characterized the frequency of circulating CXCR5^+^CD4^+^ TFH cells in 27 adult patients with MCD and 14 healthy controls. We found that the potential relationship of different subsets of TFH cells and the values of clinical measures pre- and posttreatment with different medication in MCD patients were different. Our data may provide new insights into the pathogenesis of MCD and suggest that the changes in the frequency of activated B and TFH cells may be valuable for the evaluation of therapeutic responses in MCD patients.

## 2. Materials and Methods

### 2.1. Patients

A written informed consent was first obtained from individual participants. The experimental protocol was established according to the guidelines of the Declaration of Helsinki and was approved by the Human Ethics Committee of Jilin University (Jilin University, Changchun, China). A total of 27 patients with MCD visiting clinic for the first time were recruited in the inpatient service of the Department of Nephrology, the First Hospital of Jilin University, from December 2011 to February 2013. All MCD patients were confirmed to have nephritic syndrome on the basis of the diagnostic criteria, including massive proteinuria (>3.5 g/day), hypoalbuminemia (albumin < 30 g/L), hyperlipidemia, and edema. No patient had received hormone drugs or other immunosuppressive agents in the last six months prior to this study. Renal histological diagnoses were conducted according to World Health Organization (WHO) histological classification standards. In addition to careful collection of clinical manifestations the plasma laboratory tests of antinuclear antibody (ANA), anti-Sm antibody, anti-SSA, anti-SSB, antineutrophil cytoplasmic antibody (ANCA), antiphospholipid antibody, plasma complements C3 and C4, and rheumatoid factor were performed for all the patients in the study. Renal histological and immunofluorescence results were also done to rule out patients with the potential autoimmune diseases [4]. Potential patients with bacterial infection, virus infection, diabetes, hypertension, and thromboembolism were excluded. In the control group, a total of 14 age and gender matched healthy controls were recruited at the Physical Examination Center of the same hospital and these HC had no history of any chronic disease or recent infection. The demographic and clinical data of individual participants were recorded and analyzed.

### 2.2. Treatment and Followup

The patients with 24-hour urinary protein >1 g were treated with prednisolone (PDN, Tianyao Pharmaceuticals, Tianjin China) of 1 mg/kg/day dosage for the first two months which was then gradually tapered to a maintenance dose of 10 mg/day dosage over next six-month duration. Among these patients some needed immunosuppressant along with prednisolone. In addition, the patients with high risk of hypercoagulable state were treated with dipyridamole (50 mg/d, Yunpeng Pharmaceutical, Shanxi, China). The patients were followed up monthly for 8 to 12 weeks. Blood samples were collected at the time of kidney biopsy and after 8–12 weeks of treatment.

### 2.3. Blood Sampling and Analyses

Fasting venous blood samples were collected from individual healthy controls and MCD patients. One portion of blood was used for preparing peripheral blood mononuclear cells (PBMCs) by density-gradient centrifugation using Ficoll-Paque Plus (Amersham Biosciences, Little Chalfont, UK) and the remaining blood samples were centrifuged for preparing serum samples. The numbers of leukocytes and lymphocytes were examined and the concentrations of serum triglycerides, cholesterol, uric acid, and albumin were determined using ADVIA 1650 biochemical analyzer (Bayer, Pittsburg, PA, USA). In addition, 24 h urine samples were collected from individual participants, and the concentrations of urinary proteins and microscopic hematuria were measured. The values of estimated glomerular filtration rate (eGFR) of individual participants were calculated using the revised eGFR formula [17].

### 2.4. Flow Cytometry Analysis

Human PBMCs at 10^6^/tube were stained in duplicate with PE-anti-CXCR5 (Biolegend, San Diego, USA) and APC-Cy7-anti-CD3, PerCP-anti-CD4, APC-anti-ICOS, and FITC-anti-PD-1 (Beckton Dickinson, San Jose, USA) at room temperature for 30 minutes, respectively. After being washed with PBS, the cells were subjected to flow cytometry analysis using a FACSAria II. At least 50,000 events per sample were analyzed by FlowJo software (v5.7.2) [18].

### 2.5. ELISA for Sera IL-21

The concentrations of serum IL-21 in individual MCD patients and HC were measured by ELISA using a human IL-21 ELISA kit (Roche Diagnostics, Lewes, UK), according to the manufacturer's instructions. Briefly, individual serum at 1 : 2 dilutions were subjected to ELISA analysis and the concentrations of serum IL-21 in individual samples were calculated according to the standard curve established using the recombinant IL-21 provided. The limitation of detection for human IL-21 was 11.99 pg/mL.

### 2.6. Cytometric Bead Array (CBA) Analysis of Sera Cytokines

The concentrations of sera IFN-*γ*, TNF-*α*, IL-2, IL-4, IL-10, IL-6, and IL-17A were determined by CBA [19], according to the manufacturer's protocol (BD Biosciences) with minor modifications. Briefly, individual serum (25 *μ*L/each) were tested in duplicate, as previously described [20]. The concentrations of sera cytokines were quantified using the CellQuest Pro and CBA software (Becton Dickinson) on a FACSAria II.

### 2.7. Statistical Analysis

All data are expressed as median and range. The difference between two groups was analyzed by the Kruskal-Wallis H nonparametric test. The relationship between variables was analyzed by Spearman's rank correlation test. All statistical analyses were performed by the SPSS version 19.0 software. A two-sided* P* value of <0.05 was considered statistically significant.

## 3. Results

### 3.1. A Higher Frequency of Circulating CXCR5^+^CD4^+^ TFH Cells in the MCD Patients

To determine the potential role of CXCR5^+^CD4^+^ TFH cells in the development of MCD, 27 Chinese patients with MCD visiting clinic for the first time and 14 healthy controls (HC) were recruited. There was no significant difference in the distribution of age and gender between the patients and HC (Table 1). Furthermore, there was no significant difference in leukocyte and lymphocyte count, the concentrations of serum uric acid, triglycerides, cholesterol, and albumin, and microscopic hematuria between these two groups. As expected, the concentrations of 24 h urinary proteins were significantly higher in the patients than that in the HC, but the values of eGFR in the patients were significantly less than that in the HC, suggesting that those patients had kidney function impairment.

**Figure 1:**
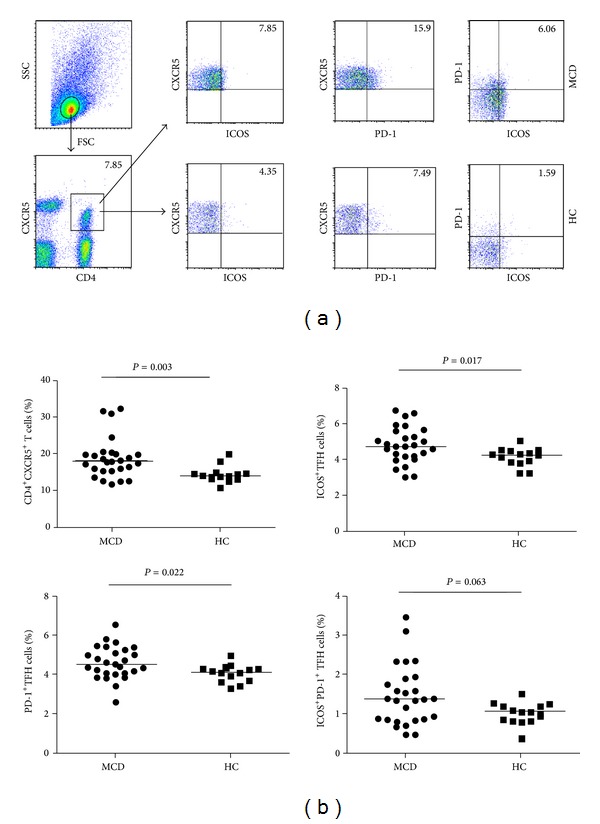
Flow cytometry analysis of TFH cells. PBMCs from MCD patients' pre- and posthormone drugs treatment as well as HC were stained with anti-CD4, anti-CD3, anti-CXCR5, anti-ICOS, and anti-PD-1. The cells were gated initially on living lymphocytes and then on CD3^+^CD4^+^ T cells. Subsequently, the frequency of CD4^+^CXCR5^+^, CD4^+^CXCR5^+^ICOS^+^, CD4^+^CXCR5^+^PD-1^+^, and CD4^+^CXCR5^+^PD-1^+^ICOS^+^ TFH cells was analyzed by flow cytometry. (a) Flow cytometry analysis and (b) quantitative analysis. Data shown are representative dot plug or expressed as the mean % of different subsets of TFH cells in total CD3^+^CD4^+^ T-cells individual subjects from two separate experiments. The horizontal lines represent the median values.

**Figure 2:**
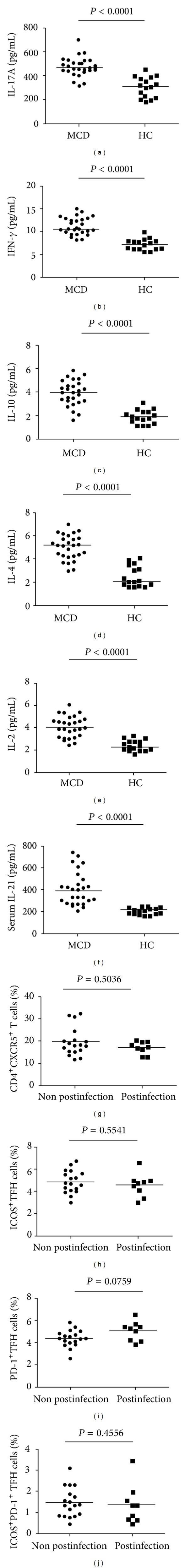
Analysis of sera cytokines in MCD patients. The difference of TFH cells subsets on postinfection and non-postinfection MCD patients. The levels of sera IL-2, IL-4, IL-10, IL-17A, and IL-21 and IFN-*γ* in individual subjects were tested by CBA and ELISA, respectively (a–f). Data are expressed as the mean values of individual samples from three separate experiments. The difference of TFH cells subsets on postinfection and non-postinfection (g–j). The horizontal lines represent the median values.

As shown in Figure 1, there was no significant difference in the numbers of circulating CD3^+^CD4^+^ T cells between the MCD patients and HC. The percentage of peripheral blood CD4^+^CXCR5^+^, CD4^+^CXCR5^+^ICOS^+^, and CD4^+^CXCR5^+^PD-1^+^ in CD3^+^CD4^+^ T cells in the patients were significantly higher than that in the HC (18.83 (11.70–32.30) versus 14.40 (10.80–19.90), *P* = 0.003; 4.78 (3.01–6.73) versus 4.12 (3.23–5.01), *P* = 0.017; and 4.59 (2.59–6.51) versus 4.04 (3.28–4.93), *P* = 0.022, resp., in Figure 1(b)). However, there were no significant differences in the frequency of circulating CD4^+^CXCR5^+^ICOS^+^PD-1^+^ TFH cells between the MCD patients and HC in this population. Then, we examined the levels of sera cytokines by CBA and ELISA. We found that the concentrations of sera IL-17A, IFN-*γ*, IL-10, IL-4, IL-2, and IL-21 were significantly higher in MCD patients than that in the HC (*P* < 0.05, Figures 2(a)–2(f)). Furthermore, we analyzed the influence of postinfection on different subsets of TFH cells. We found that there were no significant differences between infection group and no infection group. In the CD4^+^CXCR5^+^T, ICOS^+^ TFH, ICOS^+^PD^−^1^+^ TFH cells, non post infection group was higher than post infection group, but there were no significant differences (*P* = 0.5036, 0.5541, 0.4556, Figures 2(g), 2(h), and 2(j)); in the PD-1^+^ TFH cells, non post infection group was lower than post infection group, but there were no significant differences (*P* = 0.0759, Figure 2(i)). Which may indicate that post infection did not influence the level of different subsets of TFH cells in MCD patients. Together, these data clearly indicated a higher frequency of different subsets of CD4^+^CXCR5^+^ TFH cells and significantly elevated levels of sera cytokines in patients with MCD.

### 3.2. The Relationship of the Percentages of CD4^+^CXCR5^+^ with CD4^+^CXCR5^+^ICOS^+^ and CD4^+^CXCR5^+^PD-1^+^ TFH Cells and the Values of Clinical Measures in MCD Patients

**Figure 3:**
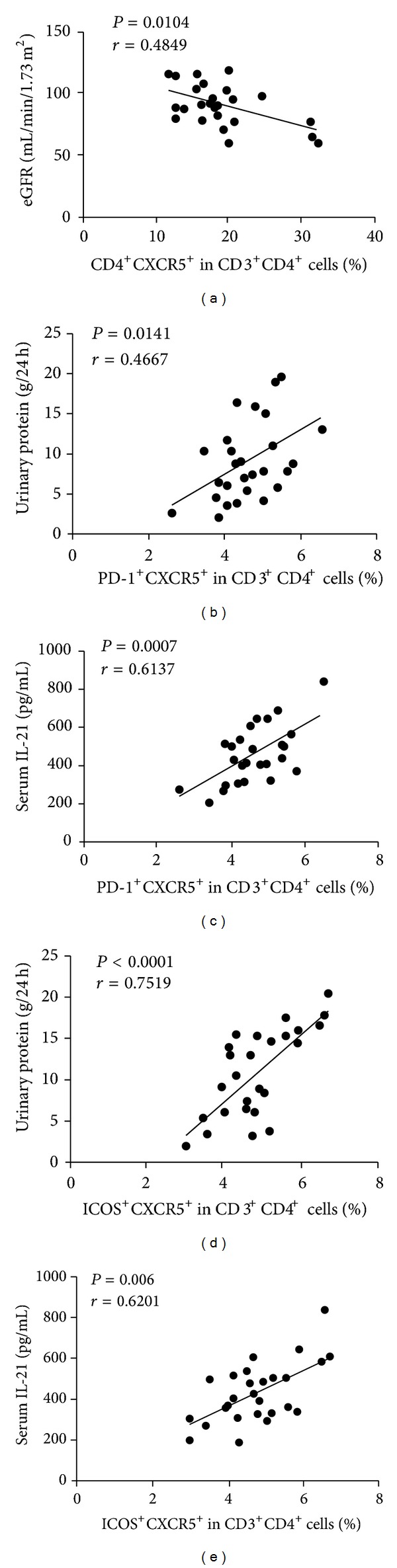
Correlation analysis of clinic pathological features of MCD with the percentages of circulating TFH cells in MCD patients. (a) The values of eGFR are negatively associated with the percentages of CD4^+^CXCR5^+^ TFH cells. (b-c) The concentrations of 24 h urinary proteins and sera IL-21 are positively correlated with the percentage of PD-1^+^CD4^+^CXCR5^+^ TFH cells. (d-e) The concentrations of 24 h urinary proteins and sera IL-21 are positively correlated with the percentage of ICOS^+^CD4^+^CXCR5^+^ TFH cells.

To understand the importance of CD4^+^CXCR5^+^ TFH cells in the pathogenesis of MCD, we analyzed the potential association of the percentages of circulating CD4^+^CXCR5^+^, CD4^+^CXCR5^+^ICOS^+^, and CD4^+^CXCR5^+^PD-1^+^ TFH cells with the values of clinical measures tested in these patients. We found the percentages of circulating CD4^+^CXCR5^+^ TFH cells were correlated negatively with the values of eGFR in these patients (*r* = −0.0104, *P* = 0.4849, Figure 3(a)). The percentages of circulating CD4^+^CXCR5^+^PD-1^+^ TFH cells were correlated positively with the concentrations of 24 h urinary proteins (*r* = 0.141, *P* = 0.4647, Figure 3(b)) and the levels of serum IL-21 (*r* = 0.0007, *P* = 0.6137, Figure 3(c)). The percentages of circulating CD4^+^CXCR5^+^ICOS^+^ TFH cells were correlated positively with the concentrations of 24 h urinary proteins (*r* = 0.7519, *P* < 0.0001, Figure 3(d)) and the levels of serum IL-21 (*r* = 0.6201, *P* = 0.006, Figure 3(e)). We also analyzed the relationship between clinical index and cytokines. We did not observe any significant correlation between clinical index (proteinuria, GFR) and cytokines (IL17-A, IFN, IL-10, IL-4, IL-2, and serum IL-21). It may indicate that MCD is only correlated with TFH cells. These data suggest that CD4^+^CXCR5^+^, CD4^+^CXCR5^+^PD-1^+^, and CD4^+^CXCR5^+^ICOS^+^ TFH responses may be associated with the pathogenesis of MCD.

### 3.3. The Values of Clinical Measures, the Frequency of CXCR5^+^CD4^+^ TFH Cells, and the Levels of Sera Cytokines in MCD Patients following Treatment

**Figure 4:**
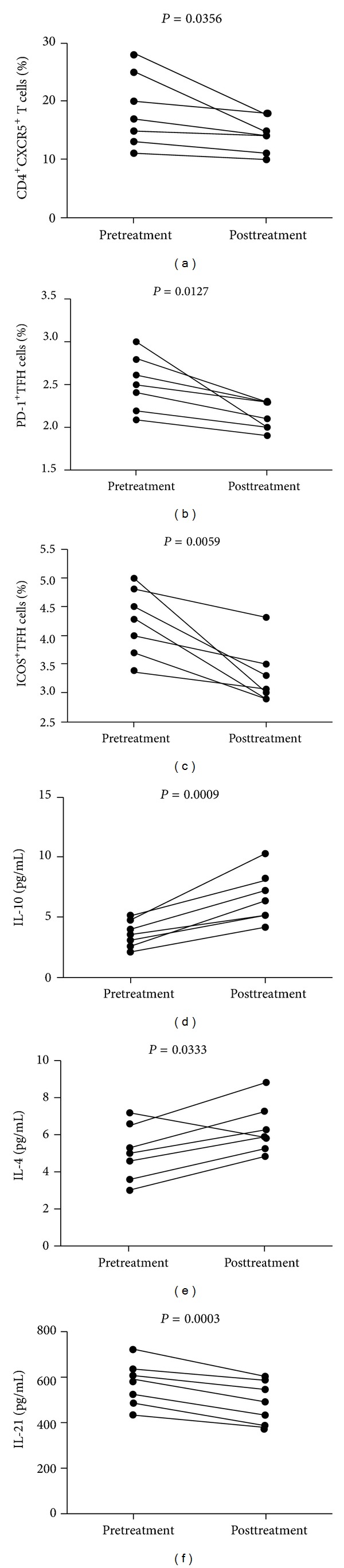
Altered frequency of TFH cells and levels of sera cytokines in MCD patients following treatment. The percentages of different subsets of TFH cells and the levels of sera cytokines were compared in MCD patients' pre- and posttreatment. Data are expressed as the mean % or concentrations of individual subjects from three separate experiments (*n* = 7 for a–f). (a) The percentages of CD4^+^CXCR5^+^ in the total CD3^+^CD4^+^ T cells in individual patients' pre- and posttreatment, (b) the percentage of CD4^+^CXCR5^+^PD-1^+^ TFH cells in total CD3^+^CD4^+^ T cells in individual patients' pre- and posttreatment, (c) the percentage of CD4^+^CXCR5^+^ICOS^+^ TFH cells in total CD3^+^CD4^+^ T cells in individual patients' pre- and posttreatment, and (d–f) the levels of IL-10, IL-4, and IL-21 in individual patients' pre- and posttreatment. The horizontal lines represent the median values.

Next, we examined the impact of treatment on the values of clinical measures, the frequency of circulating CD4^+^CXCR5^+^ TFH cells, and the levels of sera cytokines in 7 patients who were followed up for 8–12 weeks. There were altogether 7 patients with complete records and another 20 patients failed to follow up. Among those 7 patients with complete records, four patients were treated with PDN and the other three patients received immunosuppressant or dipyridamole in addition to PDN. We found that though there was no significant difference in the values of many measures, the treatment significantly reduced the concentrations of 24 h urinary protein (Table 2). In addition, although there was no significant change in the total numbers of CD3^+^CD4^+^ T cells in these patients' pre- and posttreatment, the percentages of circulating CD4^+^CXCR5^+^ (*P* = 0.0356), CD4^+^CXCR5^+^PD-1^+^ TFH cells (*P* = 0.0127), and CD4^+^CXCR5^+^ICOS^+^ TFH cells (*P* = 0.0059) were significantly reduced (Figures 4(a)–4(c)). Similarly, the levels of sera IL-10 and IL-4 in those patients after treatment were significantly higher than those before treatment (*P* = 0.0009, *P* = 0.0333, resp., Figures 4(d) and 4(e)) while the levels of serum IL-21 were significantly reduced in those patients after treatment when compared with the levels before treatment (*P* = 0.0003, Figure 4(f)). There was no significant difference in the levels of other sera cytokines tested before and after treatment. Therefore, treatment significantly improved proteinuria and reduced TFH responses but elevated inhibitory IL-10 and IL-4 responses in patients with MCD.

### 3.4. Treatment with PDN Alone Reduces the Frequency of TFH Cells and Modulates the Levels of Sera Cytokines in MCD Patients

**Figure 5:**
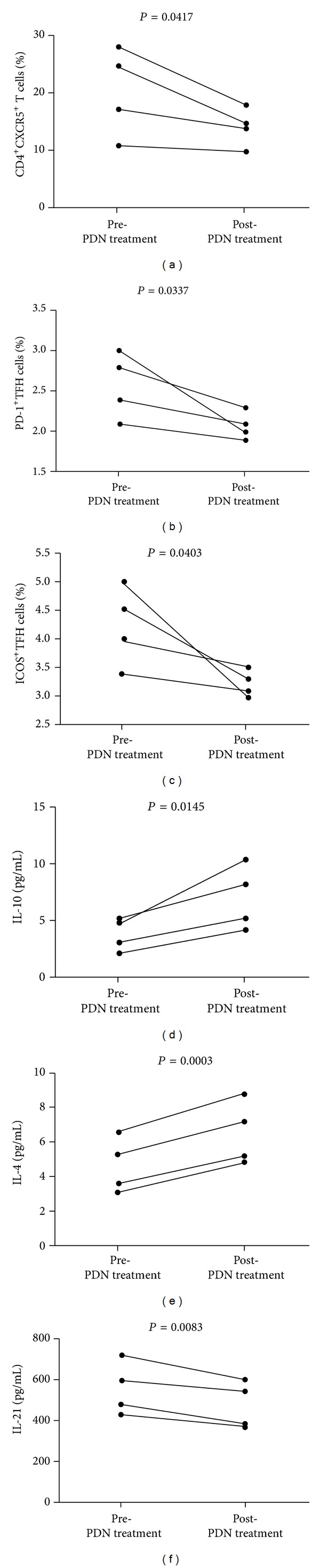
Altered frequency of TFH cells and levels of sera cytokines in MCD patients following PDN treatment. The percentages of different subsets of TFH cells and the levels of sera cytokines were compared in MCD patients' pre- and post-PDN treatment. Data are expressed as the mean % or concentrations of individual subjects from three separate experiments (*n* = 4 for a–f). (a) The percentages of CD4^+^CXCR5^+^ in the total CD3^+^CD4^+^ T cells in individual patients' pre- and post-PDN treatment, (b) the percentage of CD4^+^CXCR5^+^PD-1^+^ TFH cells in total CD3^+^CD4^+^ T cells in individual patients' pre- and post-PDN treatment, (c) the percentage of CD4^+^CXCR5^+^ICOS^+^ TFH cells in total CD3^+^CD4^+^ T cells in individual patients' pre- and post-PDN treatment, and (d–f) the levels of IL-10, IL-4, and IL-21 in individual patients' pre- and post-PDN treatment. The horizontal lines represent the median values.

To further elucidate the role of PDN in TFH cell responses, we characterized the frequency of circulating CD4^+^CXCR5^+^ TFH cells in four patients who were only treated with PDN for 8- to 12-week duration. We found that treatment not only reduced the concentration of 24-hour urinary protein but also increased level of sera albumin. However, the treatment did not significantly alter the values of other measures (Table 3). Characterization of circulating CD4^+^CXCR5^+^ T cells revealed that there was not a significant difference in the numbers of CD3^+^CD4^+^ T cells in those patients' pre- and posttreatment, but the percentage of circulating CD4^+^CXCR5^+^, CD4^+^CXCR5^+^PD-1^+^ TFH, and CD4^+^CXCR5^+^ICOS^+^ TFH cells posttreatment were significantly reduced compared to pretreatment (*P* = 0.0417, *P* = 0.0337, *P* = 0.0403, resp., Figures 5(a)–5(c)). Similarly, the levels of sera IL-10 and IL-4 were significantly elevated, while IL-21 were reduced in posttreatment with PDN (*P* = 0.0145, *P* = 0.0003, *P* = 0.0083, resp., in Figures 5(d)–5(f)). Thus, in MCD patients PDN treatment significantly improved proteinuria and inhibited CD4^+^CXCR5^+^ TFH responses but enhanced inhibitory IL-10 and IL-4 responses.

### 3.5. FACS Analysis of the Number of Different Subsets of B Cells. The Relationship between the Percentages of Different Subsets of T Follicular Helper Cells (TFH) and B Cells

**Figure 6:**
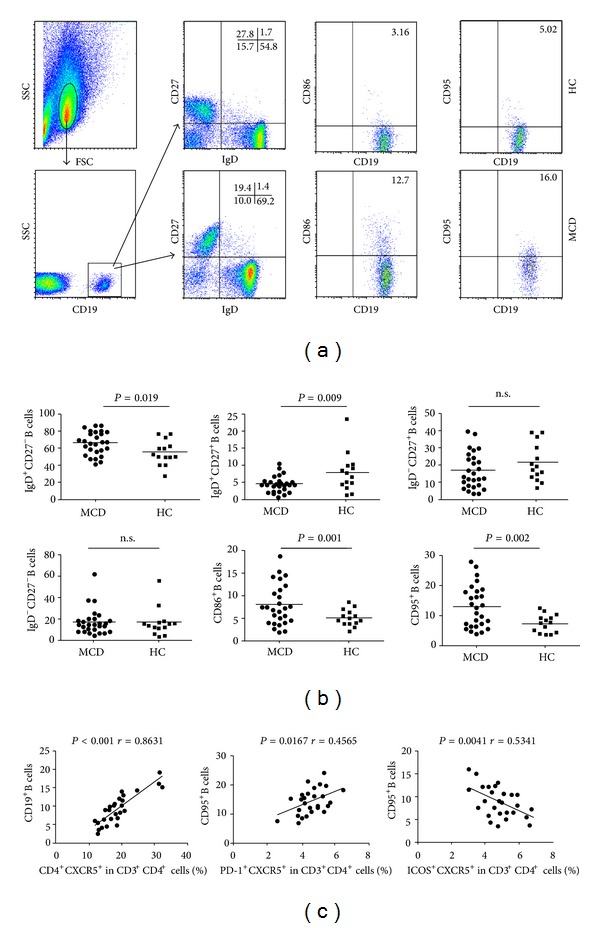
Flow cytometry analysis of B cells. The relationship of the percentages of different subsets of T follicular helper cells (TFH) with B cells in MCD patients. PBMCs from MCD patients and HC were stained with anti-CD19, anti-CD38, anti-CD95, and anti-IgD. The cells were gated initially on living lymphocytes and then on CD19^+^ B cells. Subsequently, the frequency of CD19^+^IgD^+^CD27^−^ B cells, CD19^+^IgD^+^CD27^+^ B cells, CD19^+^IgD^−^CD27^+^ B cells, CD19^+^IgD^−^CD27^−^ B cells, CD19^+^CD86^+^ B cells, and CD19^+^CD95^+^ B cells was analyzed by flow cytometry. (a) Flow cytometry analysis, (b) quantitative analysis, and (c) the percentages of different subsets of T follicular helper (TFH) and B cells. Data shown are representative dot plug or expressed as the mean % of different subsets of B cells. The horizontal lines represent the median values.

To determine the role of B cells in the pathogenesis of MCD, we characterized the frequency of different differentiation stages of B cells by flow cytometry analysis. As shown in Figure 6, the percentages of IgD^+^CD27^−^CD19^+^ (naive B), CD86^+^CD19^+^, and CD95^+^CD19^+^ B cells in those patients were significantly higher than that in the HC (*P* = 0.019, *P* = 0.001, *P* = 0.002). In contrast, the frequency of IgD^+^CD27^+^CD19^+^ preswitch memory B cells was significantly lower in the patients than that in the HC (*P* = 0.009). There was no significant difference in the frequency of IgD^−^CD27^+^CD19^+^ postswitch memory B cells and IgD^−^CD27^−^CD19^+^ double-negative B cells between the MCD patients and HC. Given that CD86 and CD95 were upregulated in B cell, our data indicated that the higher frequency of activated B cells contributed to the pathogenesis of MCD in Chinese patients with new onset MCD. To investigate the potential role of B cells in the development of MCD, we characterized relationship between the percentages of different subsets of T follicular helper (TFH) and B cells. As is shown in Figure 6(c), the percentages of CD4^+^CXCR5^+^ T cells were correlated positively with the frequency of B cells in the MCD patients (*P* < 0.001, *r* = 0.8631). The percentages of CD95^+^ B cells were correlated positively with the frequency of programmed death 1 (PD-1)^+^ TFH cells in the MCD patients (*P* = 0.0167, *r* = 0.4565). The percentages of CD95^+^ B cells were correlated negatively with the frequency of ICOS^+^ TFH cells in MCD patients (*P* = 0.0041, *r* = 0.5341).

## 4. Discussion

To understand the importance of TFH cells, we analyzed the potential association of the percentages of different types of TFH cells with the values of clinical parameters in patients in our study. TFH cells depend on expression of the master regulator transcription factor Bcl6. Distinguishing features of TFH cells are the expression of CXCR5, PD-1, IL-21, and ICOS, among other molecules, and the absence of Blimp-1. ICOS signaling is important for the maintenance and/or generation of TFH cells [21, 22] and reinforcing the association between ICOS expression and IL-10 production. PD-1 is induced by sustained TCR signaling and might act as a negative regulator of CD4^+^ T-cell proliferation. As a result, the absence of PD-1 signaling triggers a higher frequency of TFH cells [23]. IL-21 can induce its own expression in TFH cells as well as triggering a TFH cell-like state and Bcl6 expression in vitro [24, 25].

In this study, we found a significantly higher frequency of CD4^+^CXCR5^+^, CD4^+^CXCR5^+^ICOS^+^, and CD4^+^CXCR5^+^PD-1^+^ T cells in the patients than that in the HC. Furthermore, we detected significantly higher levels of serum IL-21 in those patients. More importantly, we found that the percentage of circulating CD4^+^CXCR5^+^ TFH cells were negatively correlated with the values of eGFR and the percentage of CD4^+^CXCR5^+^ PD-1^+^ CD4^+^CXCR5^+^ ICOS^+^ TFH cells were correlated positively with the levels of serum IL-21 as well as 24 h urinary proteins in those patients. These data suggest that both CD4^+^CXCR5^+^ PD-1^+^ TFH cells and CD4^+^CXCR5^+^ ICOS^+^ TFH cells may have some relationship with the pathogenesis of MCD. A recent study presented evidence that ICOS promotes TFH cell formation through enhanced IL-21 expression in a dose-dependent manner [26]. A previous study has shown that PD-1 expression on TFH cells regulates the selection and survival of plasma cells in the germinal center and promotes IL-21 production [23]. IL-21 is a crucial cytokine for the functional development of TFH cells [27].

ICOS belongs to the CD28 family and is expressed on activated T cells [28]. It plays an important role in the regulation of T-cell-dependent antibody responses and germinal-center reactions and also plays a pivotal function in TFH cell recruitment to the follicle [29]. Recently, this costimulatory pathway was also found to be important for the generation and maintenance of CXCR5^+^ TFH cells. More specifically, ICOS deficient mice showed impaired development of CXCR5^+^ TFH cells in response to primary or secondary immunization with sheep red blood cells. It has also been shown that ICOS is highly expressed by human tonsillar CXCR5^+^ T cells within the light zone of germinal centers and efficiently supports immunoglobulin production [30, 31]. Additionally, ICOS deficiency in humans and mice resulted in substantially reduced numbers of TFH cells and profound defects in B-cell maturation and immunoglobulin isotype switching, indicating an essential role for ICOS in the differentiation of TFH cells [32, 33]. Also, a previous study has shown that PD-1 expression on TFH cells regulates the selection and survival of plasma cells in the germinal center and promotes IL-21 production [23]. It is possible that CD4^+^CXCR5^+^PD-1^+^ TFH cells may provide a costimulation signal to activate antigen-specific auto reactive B cells by positively selecting auto reactive B cells and promote long-lived plasma cell development and survival, leading to high levels of autoantibody production [30]. Thereby, the percentages of circulating CD4^+^CXCR5^+^PD-1^+^ TFH cells and CD4^+^CXCR5^+^ICOS^+^ TFH cells may serve as a biomarker of MCD patients.

The underlying cause for minimal change disease (MCD) is unclear, but it is often preceded by an infection or an allergic reaction [33]. It has been proposed that MCD reflects a disorder of T lymphocytes conducive to inducing a circulating factor of immune origin altering glomerular permeability [34, 35].

The treatment of MCD in adults is challenging for several reasons. First, because so many patients respond to initial therapy as MCD is believed to have a “benign” course, there are no controlled treatment trials in adults. Second, the pathogenesis of MCD remains unknown. Third, problem in the treatment of MCD is the variability in response patterns and course of the disease [4]. Hormone drugs can inhibit inflammation by downregulating T- and B-cell function and reducing cytokine production and these drugs have been widely used for the treatment of MCD patients. We found that treatment with hormone drugs for 8–12 weeks not only significantly reduced the frequency of circulating TFH cells but also decreased the levels of serum IL-21. Recently, some researchers found that rituximab may be effective in MCD patients [36–39]. It is still not clear how B-cell depletion can induce remission in MCD. But it has been proposed that rituximab could make the effect by indirect inhibiting B cells. Their removal could have a restraining effect on other immune cells, such as T lymphocytes, dendrite cells, or macrophages [40–42]. We are interested in further investigating the treatment of MCD and the molecular mechanisms underlying the treatment in regulating the survival and function of TFH cells.

Human T lymphocytes can be divided into two groups: Th1 cells and Th2 cells. Some researchers have suggested that generation of Th-expressing Th1 (proinflammatory) or Th2 (anti-inflammatory) cytokines following priming of naive CD4^+^ T cells by Ag is influenced by the nature of TCR signaling as well as by Th1 cells producing IFN-*γ* and IL-2, whereas Th2 cells synthesize that T cells from relapse display a downregulation of the IL-12R *β*2 chain, which is compatible with Th2 polarization in MCD [43]. In addition, patients with MCD often display a defect in delayed-type hypersensitivity response, suggesting an abnormal Th1-dependent cellular immunity [44, 45]. However, recent studies have revealed that patients with MCD have a predominant ongoing T-helper cell type 2 (Th2) immune response. Therefore, MCD may be an example of a Th2-dependent glomerular disease [46]. In this study, we found that the levels of sera IL-17A, IFN-*γ*, IL-10, IL-4, and IL-2 in MCD patients were significantly higher than that in the HC, and treatment with hormone drugs significantly elevated the concentrations of sera IL-4 and IL-10 but did not affect the levels of sera IL-2, IL-6, IFN-*γ*, and TNF-*α*, associated with reduced clinical symptoms in those MCD patients. Our data suggest that proinflammatory Th1 and Th17 responses may participate in the pathogenesis of MCD.

Deregulated activation of both TFH and B cells has been associated with the pathogenic process of many autoimmune diseases in humans [47, 48]. In this study, we found that the frequency of CD19^+^IgD^+^CD27^−^ naive B cells in MCD patients was significantly higher than that in the HC, while the percentages of preswitch CD19^+^IgD^+^CD27^+^ B memory cells in MCD patients were significantly lower than that in the HC. This suggests that antigen stimulation may promote the redistribution of B cells. Activated B cells increased the expression levels of certain activation markers, such as CD86 and CD95 [49, 50]. CD86 is a critical costimulatory molecule for B-cell activation and CD95 is associated with apoptosis. In addition, we found that the percentages of ICOS^+^ TFH cells were correlated positively with the frequency of total B cells and negatively with the frequency of CD95^+^ B cells in the MCD patients. Furthermore, the percentages of PD-1^+^ TFH cells were correlated positively with the frequency of CD95^+^ B cells in those patients. Of note, the ICOS-mediated T and B cell interaction usually promotes B-cell activation, while the CD95-mediated T- and B-cell interaction commonly triggers B cell apoptosis [51]. These findings reveal that active TFH cells may regulate B-cell activation in the process of MCD.

In conclusion, our data showed that the percentages of activated B and TFH cells significantly increased in MCD patients as compared with that in the HC. Further studies are warranted to explore the roles of different subsets of B and TFH cells in the pathogenesis of MCD, to understand the mechanisms of regulation and activation of B and TFH cells, and find a new target for intervention of MCD.

## Figures and Tables

**Table 1:** The demographic and clinical characteristics of participants.

	MCD (*n* = 27)	Healthy controls (*n* = 14)
Age, year	45 (15–74)	43 (19–69)
Female/male	8/19	4/10
Urinary protein, g/24 h	9.51 (1.92–19.40)∗	0.05 (0–0.10)
Serum uric acid, *μ*mol/L	355 (198–657)	365.0 (230–400)
Triglycerides, mmol/L	2.48 (0.62–6.53)	1.21 (0.50–1.50)
Cholesterol, mmol/L	9.49 (4.47–12.83)	4.17 (2.80–5.35)
eGFR, mL/min/1.73 m^2^	82.43 (60.05–115.3)∗	98.25 (90.00–110.00)
Serum albumin, g/L	22.31 (9.81–57.7)	44.10 (40.6–49.1)
Microscopic hematuria, rbc/hpf	4.85 (1.0–16.20)	1.15 (0–2.5)
WBC, 10^9^/L	7.73 (3.30–22.99)	6.43 (4.92–9.21)
Lymphocytes, 10^9^/L	2.73 (0.83–12.90)	1.02 (0.42–1.72)

Data shown are median and range, except those specified. *P < 0.05 versus the HC.

**Table 2:** The effect of treatment on the values of clinical measures in the follow-up MCD patients.

	Pretreatment	Posttreatment
Age, years	46 (21–61)	46 (21–61)
Female/male	1/6	1/6
Urinary protein, g/24 h	8.96 (6–19.22)	3.22 (0.97–5.27)∗
Serum uric acid, *μ*mol/L	357 (198–411)	343 (227–421)
Triglycerides, mmol/L	1.81 (1.55–6.53)	1.58 (0.57–2.98)
Cholesterol, mmol/L	9.85 (6.2–11.57)	9.09 (5.67–10.45)
eGFR, mL/min/1.73 m^2^	85.24 (35.31–114.2)	83.27 (57.46–121.27)
Serum albumin, g/L	15.3 (11–33.2)	17.5 (14.3–39.2)
Microscopic hematuria, rbc/hpf	3.6 (1.1–16.2)	2.8 (0.9–14.6)
WBC 10^9^/L	8.1 (4.02–15.08)	6.03 (4.98–9.23)
Lymphocytes 10^9^/L	2.01 (0.83–4.13)	2.07 (0.53–3.95)

Data are present as median (range). *P < 0.05 versus the values before treatment.

**Table 3:** The effect of PDN treatment on the values of clinical measures in MCD patients.

	Before PDN treatment	After PDN treatment
Age, years	48 (38–62)	48 (38–62)
Female/male	1/3	1/3
Urinary protein, g/24 h	16.31 (6.00–19.22)	2.78 (1.36–5.27)∗
Serum uric acid, *μ*mol/L	365 (257–441)	349 (245–421)
Triglycerides, mmol/L	2.71 (1.55–3.06)	1.62 (1.21–2.85)
Cholesterol, mmol/L	10.45 (6.20–11.57)	8.97 (5.67–10.23)
eGFR, mL/min/1.73 m^2^	102.36 (94.85–115.3)	96.43 (74.3–113)
Serum albumin, g/L	15.0 (11–20.9)	16.72 (15.3–26.4)
Microscopic hematuria, rbc/hpf	3.2 (2.1–9.6)	1.18 (0.4–2.5)
WBC, 10^9^/L	6.9 (5.32–22.99)	5.79 (4.87–7.34)
Lymphocytes, 10^9^/L	3.14 (1.74–12.90)	1.52 (0.67–2.83)

Data are presented as median (range). *P < 0.05 versus the values before PDN treatment.
